# Aberrant gene methylation in non-neoplastic mucosa as a predictive marker of ulcerative colitis-associated CRC

**DOI:** 10.18632/oncotarget.7188

**Published:** 2016-02-04

**Authors:** Marco Scarpa, Melania Scarpa, Ignazio Castagliuolo, Francesca Erroi, Andromachi Kotsafti, Silvia Basato, Paola Brun, Renata D'Incà, Massimo Rugge, Imerio Angriman, Carlo Castoro

**Affiliations:** ^1^ Surgical Oncology Unit, Veneto Institute of Oncology IOV - IRCCS, Padova, Italy; ^2^ Department of Molecular Medicine, University of Padova, Padova, Italy; ^3^ Department of Surgery Oncology and Gastroenterology DISCOG, University of Padova, Padova, Italy; ^4^ Department of Medicine, University of Padova, Padova, Italy

**Keywords:** biomarker, ulcerative colitis, colorectal cancer, promoter methylation, APC

## Abstract

**Background Promoter:**

hypermethylation plays a major role in cancer through transcriptional silencing of critical genes. The aim of our study is to evaluate the methylation status of these genes in the colonic mucosa without dysplasia or adenocarcinoma at the different steps of sporadic and UC-related carcinogenesis and to investigate the possible role of genomic methylation as a marker of CRC.

**Results:**

The expression of Dnmts 1 and 3A was significantly increased in UC-related carcinogenesis compared to non inflammatory colorectal carcinogenesis. In non-neoplastic colonic mucosa, the number of methylated genes resulted significantly higher in patients with CRC and in those with UC-related CRC compared to the HC and UC patients and patients with dysplastic lesion of the colon. The number of methylated genes in non-neoplastic colonic mucosa predicted the presence of CRC with good accuracy either in non inflammatory and inflammatory related CRC.

**Methods:**

Colonic mucosal samples were collected from healthy subjects (HC) (*n* = 30) and from patients with ulcerative colitis (UC) (*n* = 29), UC and dysplasia (*n* = 14), UC and cancer (*n* = 10), dysplastic adenoma (*n* = 14), and colon adenocarcinoma (*n* = 10). DNA methyltransferases-1, -3a, -3b, mRNA expression were quantified by real time qRT-PCR. The methylation status of CDH13, APC, MLH1, MGMT1 and RUNX3 gene promoters was assessed by methylation-specific PCR.

**Conclusions:**

Methylation status of APC, CDH13, MGMT, MLH1 and RUNX3 in the non-neoplastic mucosa may be used as a marker of CRC: these preliminary results could allow for the adjustment of a patient's surveillance interval and to select UC patients who should undergo intensive surveillance.

## INTRODUCTION

Ulcerative colitis (UC) is a chronic inflammatory disorder involving the rectum and the colon that expose patients to an increased risk of colorectal cancer (CRC) [1]. In fact, in patients with UC, the cumulative risk of CRC is approximately 8% twenty years after the diagnosis and the cumulative rate of dysplasia is at least 25% [2, 3, 4]. Several independent risk factors for malignancy in UC patients have been identified such as duration of disease, extent of inflammation, history of concurrent primary sclerosing cholangitis and family history of CRC [5]. Pre-malignant histological alterations in UC patients are broadly referred to as dysplasia, rather than adenoma, since dysplasia is frequently not polypoid [6, 7].

Therefore, patients with UC at moderate or high risk for CRC are advised to undergo surveillance colonoscopy and biopsy, every 1 to 3 years accordingly to different guidelines, where multiple random biopsies are histologically evaluated for the presence of pre-cancerous changes (colorectal dysplasia) or CRC [8–10]. CRC screening in healthy subject is based on occult blood in the stool but in patients with UC the screening imply yearly colonoscopy with a significant higher grade of invasiveness. This highlights the need to identify markers for UC-related cancer that can be detected in the non neoplastic areas of the colon. The latter component would be of particular benefit because dysplasia or CRC may be difficult to identify endoscopically in UC, and a test that is not reliant on biopsies taken from dysplastic areas might be carried out by a simple sigmoidoscopy and thus it would be of particular interest as an adjuvant screening tool [11].

An altered methylation pattern in cancer cell genomes is a well recognized characteristic of tumor cells, and specific aberrant methylation events take place during the early stages of colorectal carcinogenesis leading to profound modifications in gene expression [12]. In particular, altered methylation status of RUNX3, MINT1, and COX-2 had been observed in the non-neoplastic sections of UC-related CRC colons as compared with that in the UC controls [11]. The aberrant methylation of H-cadherin (CDH13) commencing at an early stage of colorectal tumorigenesis frequently silences, in fact, the expression of this tumor suppressor gene in colorectal adenomas and cancers [13]. Besides germ-line mutations associated with hereditary familial adenomatous polyposis and somatic mutations in sporadic colorectal tumors, hypermethylation also provides an important mechanism underlying impaired APC function [14]. Moreover, the hypermethylation of the CpG island within the DNA-repair protein O-6-methylguanine-DNA-methyltransferase (MGMT) gene [15] and in the MLH1 gene is associated with a reduced gene expression observed in the majority of sporadic primary CRC cancers with microsatellite instability [16]. Finally, RUNX3 hypermethylation decreases TGF-β/BMP signalling in gastrointestinal cancer cells [17].

Therefore, the aim of our study is to evaluate the methylation status of these genes in the non-neoplastic (without dysplasia or adenocarcinoma) colonic mucosa at different steps of non inflammatory and UC-related carcinogenesis and to investigate the possible role of genomic methylation in the non-neoplastic colonic mucosa as a marker of UC-related CRC.

## RESULTS

### Patients characteristics

The promoter methylation status of five genes known to be associated with early stages of colon carcinogenesis was assessed in the colonic mucosa of 107 patients (Table 1). They consisted of 54 patients representative of non-inflammatory colon carcinogenesis pathway (30 healthy controls, 14 with adenoma and dysplasia, and 10 with cancer) and 53 patients with UC-related carcinogenesis (29 with UC, 14 with UC and dysplasia, 10 with UC and cancer).

**Table 1:** Real-time qPCR and methylation-specific PCR primers

A. Real-Time qPCR primers				
Gene	NCBI ref seq	Sequence 5′→3′	Ta.°C	Amplicon. bp
Dnmt1	NM_001130823	fw TACCTGGACGACCCTGACCTC	60	103
		rv CGTTGGCATCAAAGATGGACA		
Dnmt3a	NM_175629	fw GACAAGAATGCCACCAAAGC	60	190
		rv CGTCTCCGAACCACATGAC		
Dnmt3b	NM_006892	fw GGCAAGTTCTCCGAGGTCTCTG	60	113
		rv TGGTACATGGCTTTTCGATAGGA		

B. Methylation specific PCR primers				
Gene	sequence 5′→3′	Ta.°C	Amplicon. bp
CDH13 meth	Fw TCGCGGGGTTCGTTTTTCGC	66	243
	Rv GACGTTTTCATTCATACACGCG		
CDH13 unmeth	Fw TTGTGGGGTTGTTTTTTGT	55	242
	Rv AACTTTTCATTCATACACACA		
APC meth	Fw TATTGCGGAGTGCGGGTC	64	98
	Rv TCGACGAACTCCCGACGA		
APC unmeth	Fw GTGTTTTATTGTGGAGTGTGGGTT	62	108
	Rv CCAATCAACAAACTCCCAACAA		
RUNX3 meth	Fw TTACGAGGGGCGGTCGTACGCGGG	71	220
	Rv AAAACGACCGACGCGAACGCCTCC		
RUNX3 unmeth	Fw TTATGAGGGGTGGTTGTATGTGGG	64	220
	Rv AAAACAACCAACACAAACACCTCC		
MGMT meth	Fw TTTCGACGTTCGTAGGTTTTCGC	64	81
	Rv GCACTCTTCCGAAAACGAAACG		
MGMT unmeth	Fw TTTGTGTTTTGATGTTTGTAGGTTTTTGT	64	93
	Rv AACTCCACACTCTTCCAAAAACAAAACA		
MLH1 meth	Fw ACGTAGACGTTTTATTAGGGTCGC	56	115
	Rv CCTCATCGTAACTACCCGCG		
MLH1 unmeth	Fw TTTTGATGTAGATGTTTTATTAGGGTTGT	56	124
	Rv ACCACCTCATCATAACTACCCACA		

### DNA methyltransferase mRNA expression along colonic carcinogenesis pathway

**Figure 1:**
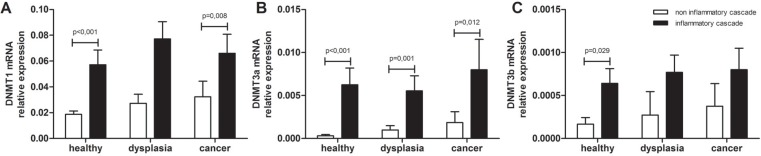
DNA methyltransferases mRNA levels in colon carcinogenesis (**A**) DNMT1 (**B**) DNMT3a and (**C**) DNMT3b mRNA levels were quantified by Real Time qPCR in the colonic mucosa specimens of patients with non-inflammatory colon carcinogenesis and UC-related colon carcinogenesis; mRNA mean levels ± SEM in the different patients groups are shown. Kruskal-Wallis ANOVA test was performed to compare multiple groups. *HC*. healthy control; *DYS*. dysplasia and adenoma; *UC*. ulcerative colitis.

To understand how the inflammatory condition may influence the genomic methylation status, Dnmt-1, -3a, and -3b mRNA expression was quantified in the different steps of non inflammatory and inflammatory carcinogenesis in colonic tissue without lesions. As shown in Figure 1, the expression of all three Dnmts tended to increase progressively along the carcinogenesis pathways; moreover a significant higher expression was detected in the UC-related carcinogenesis compared to the non-inflammatory type. Dnmt-1: healthy control (HC) vs UC patients, *p* < 0.001; adenoma vs UC and dysplasia, *p* = 0.09; adenocarcinoma vs UC and cancer, *p* = 0.008 (Figure 1A). Dnmt-3A: HC vs UC patients, *p* < 0.001; adenoma vs UC and dysplasia, *p* = 0.001; adenocarcinoma vs UC and cancer, *p* = 0.012 (Figure 1B). Dnmt-3B: HC vs UC patients, *p* = 0.029; adenoma vs UC and dysplasia, *p* = 0.288; adenocarcinoma vs UC and cancer, *p* = 0.321 (Figure 1C).

### Methylation status of gene promoters involved in the early stages of colon carcinogenesis

**Figure 2:**
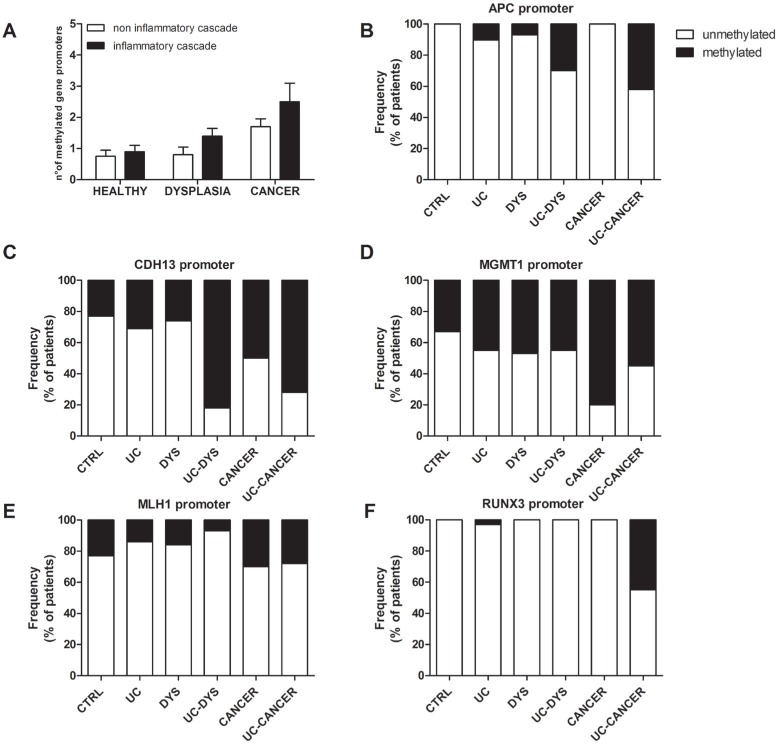
Methylation status of gene promoters involved in the early stages of colon carcinogenesis (**A**) The frequency of total methylation. defined as the sum of the methylated genes detected (range 0–5) among the different patients groups is shown. (**B**, **C**, **D**, **E**, **F**) The methylation status of APC. CDH13. MGMT. MLH1 and RUNX3 gene promoters was assessed by methylation specific-PCR of colonic mucosa specimens with no neoplastic lesion derived from patients with non-inflammatory colon carcinogenesis and UC-related colon carcinogenesis. The frequency of methylation of each gene among the different patients groups is shown. *HC*. healthy control; *DYS*. dysplasia and adenoma; *UC*. ulcerative colitis.

As shown in Figure 2A, in colonic tissue without lesions, the number of methylated genes resulted significantly higher in patients with adenocarcinoma than in HC and in those with adenomas (*p* = 0.03). Similarly, the number of methylated genes resulted significantly higher in patients with UC-related adenocarcinoma than in those with UC and in those with UC and dysplasia (*p* = 0.02). In colonic tissue without lesions, as shown in Figure 2B, the promoter regions of APC was significantly more frequently methylated in patients with UC and cancer compared to patients with non inflammatory adenocarcinoma (*p* = 0.05). Moreover, as shown in Figure 2C, the promoter regions of CDH13 was significantly more frequently methylated in patients with UC and dysplasia compared to those with inflammatory adenoma (*p* = 0.01). In contrast, the frequency of methylation of the MGMT and MLH1 promoters was not significantly different between the UC-related and the not–inflammatory carcinogenesis pathways (Figure 2D and 2E). On the contrary, as shown in Figure 2F, the promoter regions of RUNX3 was significantly more frequently methylated in patients with UC and cancer compared to patients with non inflammatory adenocarcinoma (*p* = 0.01).

### Accuracy of the number of methylated genes in predicting the presence of cancer in the colon

**Figure 3:**
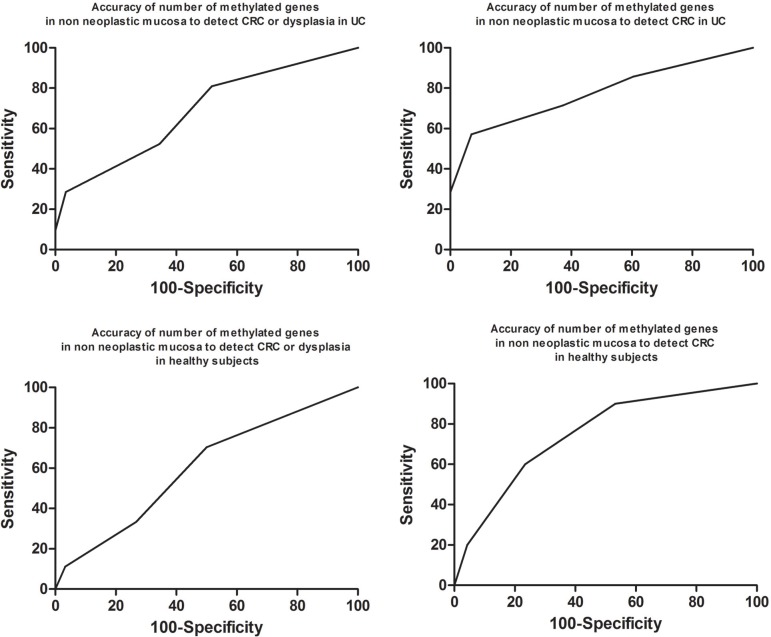
Accuracy of the number of methylated genes in predicting the presence of cancer in the colon (**A**) ROC curve showing the accuracy of the number of methylated genes in colonic mucosa without lesions in predicting the presence of dysplasia or cancer in patients UC. (**B**) ROC curve showing the accuracy of the number of methylated genes in colonic mucosa without lesions in predicting the presence of cancer in patients UC. (**C**) ROC curve showing the accuracy of the number of methylated genes in colonic mucosa without lesions in predicting the presence of adenoma or cancer in patients without UC. (**D**) ROC curve showing the accuracy of the number of methylated genes in colonic mucosa without lesions in predicting the presence of cancer in patients without UC.

To assess the accuracy of the number of methylated genes as predictor of dysplasia and cancer in inflamed and in normal colon we performed ROC curve analysis. As shown in Figure 3A, in UC-related carcinogenesis the number of methylated genes in colonic mucosa without lesions predicted the presence of dysplasia or cancer in UC patients with low accuracy (AUC = 0.68 95% CI = 0.54–0.80; *p* = 0.01833). However, the number of methylated genes in colonic mucosa without lesions predicted the presence of cancer in patients with UC with good accuracy (AUC = 0.77 95% CI = 0.63–0.88; *p* = 0.011) (Figure 3B). Indeed, over 2 methylated genes in non-neoplastic mucosa predicted the presence of CRC in patients with UC with a sensitivity of 57.1% (95% CI = 18.8–89.6) and a specificity of 93.1 (95% CI = 80.9–98.5). Similarly, as shown in Figure 3C, in non inflammatory carcinogenesis the number of methylated genes in colonic mucosa without lesions predicted the presence of adenoma or cancer with poor accuracy (AUC = 0.60 95% CI = 0.46–0.73; *p* = 0.1833). Nevertheless, the number of methylated genes in colonic mucosa without lesions predicted the presence of cancer in patients without UC with good accuracy (AUC = 0.75 95% CI = 0.62–0.85; *p* = 0.008) (Figure 3D).

### Number of methylated genes as risk factor for CRC in UC

**Figure 4:**
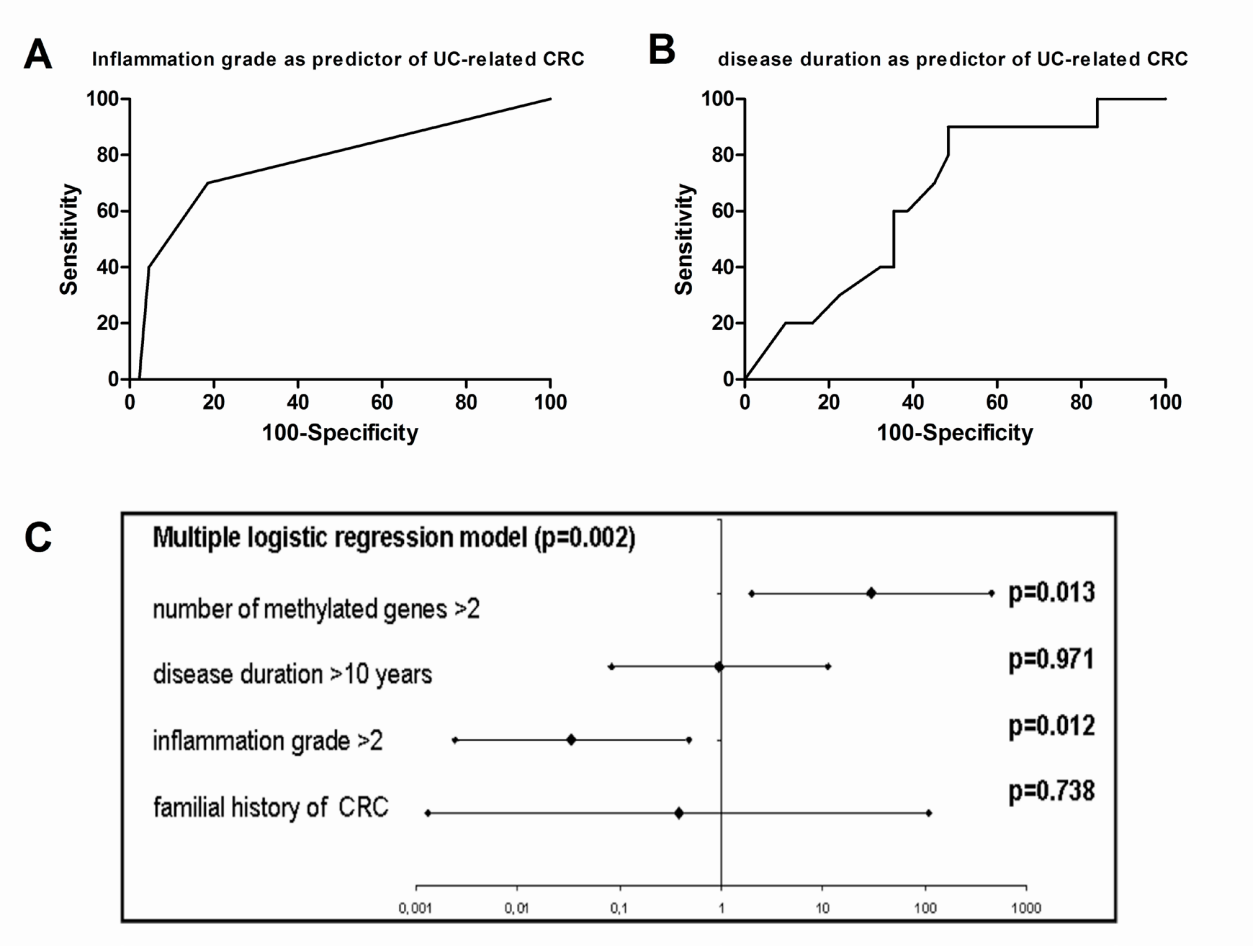
Number of methylated genes as risk factor for CRC in UC (**A**) ROC curve showing the accuracy of inflammatory grade as measured by Floren score in predicting UC-related CRC. (**B**) ROC curve showing the accuracy of disease duration of patients in predicting UC-related CRC. (**C**) Forrest plot showing a multiple logistic regression analysis including Floren score, methylation score, disease duration and familial history of CRC as possible predictors of UC-related CRC.

To assess the possible interaction with other known predictors of CRC we tested the number of methylated genes with the grade of histological inflammation, the duration of the disease and the familial history of CRC. As shown in Figure 4A, inflammatory grade as measured by Floren score on the same mucosal sample showed a good accuracy in predicting UC-related CRC (AUC = 0.77 95% CI = 0.64–0.88; *p* < 0.001). Moreover, as shown in Figure 4B, disease duration of patients showed a low accuracy in predicting UC-related CRC (AUC = 0.65 95% CI = 0.49–0.79; *p* < 0.151). Similarly, familial history of CRC showed a odd ratio of 0.578 (95% CI = 0.059–5.631) for CRC in UC. As shown in Figure 4C, multiple logistic regression analysis showed that both Floren score and methylation score, but not disease duration and familial history of CRC, were independent predictors of UC-related CRC.

## DISCUSSION

UC is a chronic inflammatory disorder that shows increased risk of CRC [1] with a cumulative risk approximately 8% twenty years after the diagnosis [2, 3, 4]. Therefore, patients with UC at moderate or high risk for CRC are advised to undergo frequent surveillance colonoscopy with multiple random biopsies [8–10]. The identification of markers for UC-related cancer that can be detected in the non neoplastic areas of the colon would greatly simplify the clinical practice and reduce patients' stress [11]. An altered methylation pattern of APC, CDH13, MGMT, MLH1 and RUNX3 genes is a well recognized characteristic of CRC tumor cells [12]. Therefore, the aim of our study is to evaluate the methylation status of these gene in the non-neoplastic colonic mucosa at the different steps of not inflammatory and UC-related carcinogenesis and to investigate the possible role of genomic methylation in the non-neoplastic colonic mucosa as a marker of UC-related CRC.

Several CpG-island-associated genes involved in cell growth control or metastasis can become hypermethylated and silenced in tumors [25]. Among these APC [13, 26], Cadherin [14, 27], MGMT [15, 28], MLH1 [16, 29] and RUNX3 [17]. The main original observation in our study is that these genes may become methylated even in the non-neoplastic mucosa of patients with CRC (UC-related or not). Moreover, some of them, such as APC and RUNX3 were significantly more frequently methylated in patients with UC and cancer compared to patients with non inflammatory adenocarcinoma and CDH13 was more frequently methylated in patients with UC and dysplasia compared to those with adenoma. These observations are very likely due to the higher expression of Dnmts in all the carcinogenesis steps in patients with UC compared to those without inflammation in their colon. During chronic inflammation. intrinsic mediators of inflammatory responses, such as proinflammatory cytokines and reactive oxygen and nitrogen species, can induce genetic and epigenetic modifications, including point mutations, deletions, duplications, recombinations, and methylation of some tumor-related genes through various mechanisms.

Dnmt-1, -3a, and -3b mRNA expression was quantified to understand how the inflammatory condition may influence the genomic methylation status in the non-neoplastic colonic mucosa. In our series, the expression of Dnmts 1 and 3A showed a significant higher expression in each steps of the UC-related carcinogenesis compared to the non-inflammatory corresponding steps. Although the mechanism responsible for increased expression of DNMT1 in UC is not well known, a previous report of DNMT1 protein overexpression in bladder cancer suggested that the mRNA expressions of DNMT1 we observed in UC may be attributable to increased cell proliferation caused by inflammation [23–26].

In fact, in our series, in non-neoplastic colonic mucosa, the number of methylated genes resulted significantly higher in patients with CRC and in those with UC-related CRC compared to the HC and UC patients and patients with dysplastic lesion of the colon. This observation has two direct implications. The first one is that in patients with high DNMTs activity, such as patients with UC, possible inhibitory agents might be tested to reduce the risk of CRC onset. The second and more direct one is that the number of methylated genes in non-neoplastic mucosa may be used in the CRC surveillance in UC patients. In fact, according to some investigators, methylation of some genes such as RUNX3, MINT1, and COX-2 can be considered potential biomarkers to detect signs of CRC in samples taken from inflamed areas of colon in UC patients [11]. Therefore, we assessed the accuracy of the number of methylated genes as predictor of CRC in inflamed and in normal condition and we concluded that either in UC or in not inflammatory condition the number of methylated genes in colonic mucosa without lesions predicted the presence of CRC with good accuracy. These data suggested a practical application for UC patients since in their case CRC surveillance is currently conducted with regular colonoscopies [30] while for healthy subjects CRC screening program is currently based on fecal blood test. In UC patients a simple sigmoidoscopy might provide adequate mucosal samples for assessing CRC risk with the advantage of a much easier bowel preparation and a shorter and less difficult endoscopic exam. Furthermore, the test has a high specificity (that measures the proportion of negatives that are correctly identified as such) making it an ideal first line test (true positive may be later assessed at second line test, in this case by a full colonoscopy with random biopsies). Indeed, the accuracy of the test might be improved with the histological grade of inflammation on the same mucosa sample as shown by multivariate analysis. Finally a further step in the clinical application of these observation might be the research of the methylation status of these genes in the stools. In fact, methylation of BMP3, vimentin, EYA4 and NDRG4 has been found to be highly discriminant in detecting signs of CRC in stool specimens [31].

In conclusions, although further longitudinal studies with large numbers of subjects are needed to clarify the role of methylation status of APC, CDH13, MGMT, MLH1 and RUNX3 in non-neoplastic mucosa as a marker of UC-associated CRC, and to validate the predictive power and clinical value of analysis of the number of methylated genes, these preliminary results could allow for the adjustment of a patient's surveillance interval and to select UC patients who should undergo intensive surveillance.

## MATERIALS AND METHODS

### Patients

A prospective cohort study of healthy controls (*n* = 30) and patients (*n* = 77) who underwent colonoscopy for screening or post-operative follow-up, colonic resection for colorectal cancer, or restorative proctocolectomy for ulcerative colitis (UC) was designed. No patients was also affected by primary sclerosing cholangitis. Biopsy samples of healthy mucosa (*n* = 30), of normal and diseased mucosa from patients with dysplastic adenoma (*n* = 14), and from patients with colon adenocarcinoma (*n* = 10) were collected. Biopsy samples of the diseased mucosa of patients with UC (*n* = 29), of those with UC and dysplasia (*n* = 14), and of those with UC and cancer (*n* = 10) were also collected. The study, which received institutional review board (Ethical Committee of the Veneto Institute of Oncology) approval (project MICCE1 IOV 2011/53), was performed according to the principles of the Declaration of Helsinki, and all those participating signed informed consent forms.

### Histopathology

Sections (3 μm) from formalin-fixed and paraffin-embedded specimens were stained with hematoxylin-eosin. Histological inflammation was quantified and classified using Floren's score [18] while the Vienna classification of gastrointestinal epithelial neoplasia was used to classify the different step of the colonic carcinogenesis [19].

### Gene expression analysis

Total RNA was extracted using the RNeasy Plus Kit (Qiagen) according to the manufacturer's protocol. At that point 0.5 μg total RNA was converted to cDNA using the Applied Biosystems cDNA Synthesis kit, again, according to the manufacturer's instructions. DNA methyltransferase-1, -3a, -3b (Dnmt-) mRNA expression was quantified by real time qRT-PCR. Specific mRNA transcripts were quantified with SYBR Green PCR Master Mix in a ABI PRISM 7000 Sequence Detection System (Applied Biosystems). Actab expression was used as reference gene for normalization. Primer sequences and PCR conditions are outlined in Table 1.

### Methylation specific PCR

Genomic DNA was extracted from tissues using a DNeasy Blood & Tissue Kit (Qiagen) according to the manufacturer's directions. Sodium bisulfate modification of gDNA was performed using the EZ DNA Methylation-Gold Kit (Zymo Research) following the manufacturer's instructions. The primers for APC, CDH13, MGMT, MLH1 and RUNX3 methylation-specific PCR and PCR conditions are outlined in Table 2. The EpiTect PCR Control DNA Set (Qiagen) was used as the positive control for the methylated and unmethylated genes. Each PCR was done in a final volume of 25 μL containing 10 ng of bisulfite converted gDNA, 1 × PCR buffer, 0.25 mmol/L deoxynucleotide triphosphate, 400 nmol/L each primer, and 1 unit ZymoTaq (Zymo Research). PCR amplification was done as follows: 95°C for 10 min followed by 40 cycles at 95°C for 30 s, the specific annealing temperature for each gene for 30 s and 72°C for 30s; and, in a final extension step, at 72°C for 7 min. PCR products were resolved by 3% agarose gel electrophoresis and each case was scored as methylated or unmethylated. Since we aimed to investigate the role of methylation we restrictively considered promoter methylation present only if the ratio between methylated/unmethylated band signal was >1. The patients' global methylation scores were calculated by summing the number of methylated genes (range 0–5).

**Table 2:** Patients characteristics

Non-inflammatory carcinogenesis	Healthy controls	Adenoma and dysplasia	Cancer
Patients (n)	30	14	10
median age (range)	59.5 (52–69) years	61 (51–69) years	63 (49–74) years
gender (male/female)	14:16	7:7	7:3
procedures	colonoscopy: 30	colonoscopy: 12	colonic resection: 8
		RPC: 2	rectal resection: 2
carcinogenesis stage	NA	LGD: 9	T1N0M0: 1
		HGD: 5	T2N0M0: 2
			T3N0M0: 2
			T3N1M0: 4
			T3N1M1: 1

UC-related carcinogenesis	UC	UC and dysplasia	UC and cancer
Patients (n)	29	14	10
median age (range)	44 (16–70) years	55 (24–72) years	54 (33–67) years
gender (male/female)	15:14	7:7	6:4
procedures	RPC: 29	RPC: 14	RPC: 9
			proctectomy: 1
carcinogenesis stage	NA	LGD: 10	T1N0M0: 8
		HGD: 4	T2N0M0: 1
			T4N1M1: 1

HGD. high grade dysplasia; LGD. low grade dysplasia; RPC. restorative proctocolectomy

### Statistical analysis

Data are shown as mean ± SEM. Non-parametric Mann–Whitney's *U*-test was carried out to compare independent variables, the Wilcoxon test was used to compare matched variables, and the Kruskal-Wallis ANOVA was performed to compare multiple variables. Receiving operator characteristics (ROC) were assessed by curve analysis as described by Henderson (21). Logistic regression modeling was undertaken to determine whether the grade of histological inflammation as measured with Floren score and the number of methylated genes were independent predictor of UC-related CRC. Both score were dichotomized on the threshold values obtained at ROC curve analysis. Statistical significance was indicated by *p* < 0.05.

## Abbreviations

APC: Adenomatous Polyposis Coli
CDH13: H-cadherin
CRC: colorectal cancer
DNMT: DNA methyltransferase
MGMT: O-6-methylguanine-DNA methyltransferase
MLH1: MutL homolog 1
RUNX3: Runt-related transcription factor 3
UC: Ulcerative Colitis
